# The Pathogenesis of Diabetes

**DOI:** 10.3390/ijms24086978

**Published:** 2023-04-10

**Authors:** Huiqin Guo, Haili Wu, Zhuoyu Li

**Affiliations:** 1 Institute of Biotechnology, The Key Laboratory of Chemical Biology and Molecular Engineering of Ministry of Education, Shanxi University, Taiyuan 030006, China; 2 College of Life Science, Shanxi University, Taiyuan 030006, China

**Keywords:** diabetes, signal pathway, insulin resistance, hyperglycemia, long-chain non-coding RNA (lncRNAs)

## Abstract

Diabetes is the most common metabolic disorder, with an extremely serious effect on health systems worldwide. It has become a severe, chronic, non-communicable disease after cardio-cerebrovascular diseases. Currently, 90% of diabetic patients suffer from type 2 diabetes. Hyperglycemia is the main hallmark of diabetes. The function of pancreatic cells gradually declines before the onset of clinical hyperglycemia. Understanding the molecular processes involved in the development of diabetes can provide clinical care with much-needed updates. This review provides the current global state of diabetes, the mechanisms involved in glucose homeostasis and diabetic insulin resistance, and the long-chain non-coding RNA (lncRNA) associated with diabetes.

## 1. Status and Characteristics of Global Diabetes

According to the latest data released by the International Diabetes Federation (IDF) [1], the global prevalence of diabetes reached 10.5% in 2021. Of these cases, 537 million adults live with diabetes, which is an increase of 16% (74 million) from 2019. However, nearly half (44.7%) of adults have not yet been diagnosed. The IDF predicts that by 2045, 784 million adults will have diabetes, which is more than double the estimated population (20%) over the same period [1,2]. Diabetes is a chronic disease characterized by hyperglycemia, which results from a relative or absolute deficiency of insulin [3], the decreased sensitivity of target cells to insulin, and the glycolipid and protein metabolism disorders [4]. More than 90% of diabetic patients suffer from type 2 diabetes [5]. People with diabetes usually exhibit four metabolic abnormalities: abnormal insulin action, insulin secretion dysfunction [6,7], increased endogenous glucose output [8], and obesity [9]. Currently, diabetes also has a high mortality rate, which has a serious threat to individuals and society [10,11].

## 2. Diabetes Triggered by Inflammation

Chronically high glucose levels in diabetic patients can lead to inflammatory dysregulation. Lekva et al. showed that high glucose activated toll-like receptor 4 (TLR4) and promoted the expression of pro-inflammatory factors, which, in turn, enhanced insulin resistance [12]. High levels of tumor necrosis factor-α (TNF-α) in the adipose tissue of obese mice resulted in insulin resistance by inhibiting peroxisome-proliferator-activated receptor γ function [13,14]. TNF-α also induced c-Jun N-terminal kinase (JNK) activation, which inhibited the insulin signaling pathway by phosphorylating insulin receptor substrate 1 (IRS-1) and decreasing glucose transporter 4 (GLUT-4) expression [15]. In addition, nuclear factor kappa-B (NF-κB) was also activated in several tissues of type 2 diabetic patients and played a central role in promoting tissue inflammatory responses [16]. NF-κB was activated in pancreatic β-cells through the action of glucose and IL-1β, and the inhibition of NF-κB protected β-cells from various types of damage, including glucotoxicity [16]. A study by Tian et al. showed that in diabetes, NF-κB usually occurs nuclear heterotopic and promotes the expression of inflammation-related factors such as TNF-α, interleukin-1β (IL-1 β), and interleukin-6 (IL-6) [17]. Panahi et al. reported that high glucose exacerbates reactive oxygen species (ROS) production and activates mitogen-activated protein kinase (MAPK) and NF-κB signaling pathways, thus inducing inflammation [18]. It has also been reported that T-cell-derived transforming growth factor-β (TGF-β), interferon γ (IFN-γ), granulocyte-macrophage colony-stimulating factor (GM-CSF) and interleukin-2 (IL-2) cytokines are involved in the immune-mediated destruction of diabetic β cells, leading to insulin resistance [19]. Jennifer et al. found that a significant elevation of TNF-α and IL-6 in diabetic patients promotes suppressors-of-cytokine-signaling 3 (SOCS3) expression [20]. Sireesh et al. reported that the activation of NRF2, a master regulator of redox homeostasis, protected pancreatic β-cells from various injuries, thereby maintaining glucose homeostasis and increasing insulin sensitivity [21]. The level of the antioxidant gene downstream of nuclear factor erythroid 2-related factor 2 (NRF2) was low in diabetic patients, and NRF2 knockout mice were highly susceptible to oxidative and inflammatory damage [21]. Plasma NRF2 was positively correlated with IL-4 and interleukin-13 (IL-13) and negatively correlated with IFN-γ and TNF-α. NRF2 activators exerted anti-diabetic effects by attenuating oxidative and inflammatory stress [21]. These results suggest that type 2 diabetes is an inflammatory disease.

## 3. Autophagy Prevented Diabetes and Its Complications

Autophagy is associated with the development of diabetes and its complications. Autophagy may further influence the development of type 1 diabetes by affecting the function of T cells. The activation of the mTOR signaling pathway and the inhibition of AMPK were found in a streptozotocin (STZ)-induced type 1 diabetes rat model, demonstrating that autophagy was inhibited [22]. Studies have shown that autophagy is essential for maintaining the structure and function of pancreatic beta cells. Jung et al. found that after the specific knockout of autophagy gene Atg7 in mouse islet beta cells, the beta cells contained a large amount of ubiquitin protein and overexpressed p62, the number of beta cells was significantly reduced, the secretion of insulin was decreased, and the mitochondria and rough endoplasmic reticulum were swollen [23]. These results indicate that beta autophagy is a protective mechanism for removing toxic ubiquitin protein, damaged cells, and p62. Metformin activated the inhibited AMPK pathway in the diabetic heart, upregulated autophagy, and exerted a protective effect [24,25]. Metformin also enhanced AMPK phosphorylation and attenuated mTOR activity, ameliorating high-glucose-induced renal tubular hypertrophy and STZ-induced diabetic kidney injury [25,26]. The deactivation of AMPK may inhibit autophagy and participate in the development of diabetic nephropathy, while the activation of AMPK improves its regulation of autophagy and alleviates diabetic kidney injury. Deletion of podocyte autophagy gene Atg5 caused glomerular lesions in aging mice and accompanied by intracellular oxidation, the aggregation of ubiquitinated proteins, and endoplasmic reticulum stress, ultimately leading to podocyte loss [27]. The key upstream regulators of autophagy, such as mTOR, are involved in the pathogenesis of diabetic nephropathy. In mice, it was shown that intervention with rapamycin (an mTOR inhibitor) inhibited the development of diabetic nephropathy by enhancing autophagy in podocytes [28]. Knockout of the tsc1 gene (which negatively regulates the mTOR factor) in mice resulted in increased endoplasmic reticulum stress and eventually diabetic nephropathy [29]. The inhibition of mTORCl activity by a very low protein diet in diabetic, adipose Wistar rats also reversed autophagic damage in Proximal Tubular Epithelial Cells (PTEC), thus ameliorating renal tubular cell injury, inflammation, and interstitial fibrosis [30]. This suggests that mTOR is necessary for maintaining sufficient cellular integrity. In the nervous system, autophagy is a clearance mechanism that removes or attenuates damage caused by cellular stress. The activation of autophagy in neural tissues is also regulated by immune factors, and in normal humans, natural auto-anti-Fas antibodies are present. Increased levels of LC3-II and Fas-related death proteins were observed in neuroblastoma SH-SY5Y cells cultured with serum from patients with neuropathy, suggesting that autophagy may play a protective role in apoptosis through the Fas-dependent pathway that activates autophagy in cells, and that Fas (CD35)-FADD-caspase-8 is associated with apoptosis [31]. In conclusion, many studies have shown that autophagy plays a positive role in the pathophysiology of diabetes. Autophagy may be a key target for the prevention and treatment of diabetes and its complications.

## 4. Glucose Homeostasis

Glucose homeostasis is the maintenance of blood glucose concentration in a relatively stable range through a series of body regulations [32]. It depends on the communication network comprising various hormones and neuropeptides released by the brain, pancreas, liver, and intestinal organs as well as fat and muscle tissues. The imbalance of glucose homeostasis leads to metabolic disorders.

The pancreas is an important participant in glucose homeostasis [33]. The interaction between insulin and glucagon is a crucial process that makes it possible for the pancreas to maintain blood glucose levels within a quite narrow range of 4–6 mM [34]. In the postprandial state, β cells stimulated by increased glucose levels secrete insulin to lower blood sugar levels [35,36]. Insulin inhibits the decomposition of liver glycogen, stimulates glycogen synthesis, converts glucose into fatty acids and triglycerides, and facilitates glucose uptake by peripheral tissues [37]. Insulin also promotes glucose uptake by muscle and fat by stimulating the translocation of glucose transporter 4 to the cell surface [38]. During fasting, when the blood glucose level is too low, α cells will release glucagon, which promotes the decomposition of liver glycogen and drives hepatic and renal gluconeogenesis [39]. In addition, glucagon restrains glycogen synthesis by inhibiting glycogen synthase in the liver and regulates the activity of phosphofructokinase 1 (PFK1) to reduce glycolysis [40,41]. The impairment of any link may lead to a disruption in glucose homeostasis, resulting in insulin resistance and ultimately developing into type 2 diabetes [42].

The liver also plays an indispensable role in regulating glucose homeostasis [43]. It maintains constant blood glucose through glycogen synthesis, glycogenolysis, glycolysis and gluconeogenesis, and has bidirectional regulation effects on glucose metabolism [44]. In the postprandial state, due to the decrease in glucagon and the increase in insulin, the liver undergoes glycogen synthesis and glycolysis to reduce glucose levels. In a state of human hunger and (or) a low blood sugar level, with diminished insulin and augmented glucagon, the liver will form glucose via glycogenolysis and gluconeogenesis to improve blood levels from a net uptake pattern to output. Meanwhile, hepatic glucose-6-phosphate is also a pivotal substance for glucose metabolism through which glucose interconverts with other monosaccharides, including fructose, galactose, and mannose, to regulate glucose levels [45].

In addition to the regulation of communication between organs, many other factors affect glucose homeostasis, such as the proportion of metabolites produced by intestinal flora [46] and gender [47]. Numerous studies have shown that intestinal dysbiosis reduces the synthesis of short-chain fatty acids, which, in turn, disturbs the intestinal barrier integrity [48,49], pancreatic β cell proliferation [50], and insulin biosynthesis [51]. Intestinal dysbiosis also influences the production of other metabolites, including branched amino acids, trimethylamine, primary bile acids, and secondary bile acids, disrupting glucose homeostasis [52,53]. Franck Mauvais-Jarvis found that men and women have regulate glucose balance differently, which may be related to sex hormones. Both men and women showed significant differences in a glucose tolerance test and insulin sensitivity [47].

## 5. The Role of the Liver in Regulating Glucose Metabolism

**Figure 1:**
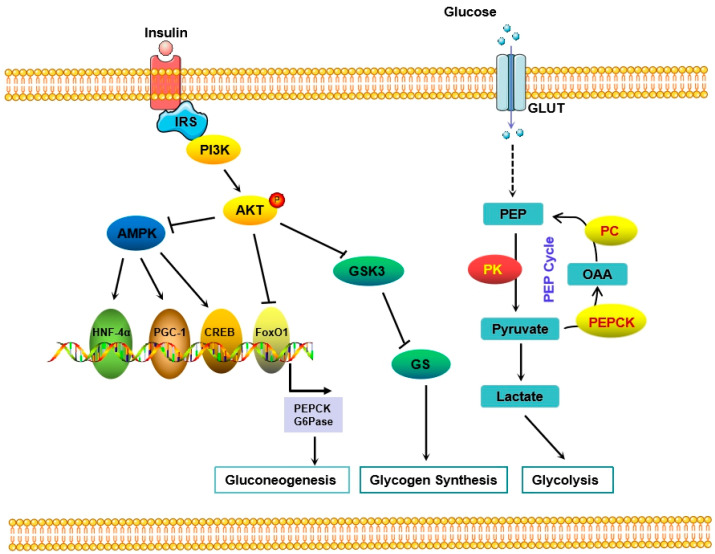
Signaling mechanisms in regulating glucose metabolism.

Recently, an increasing number of studies have believed that an abnormal glucose metabolism is the central link to hyperglycemia in diabetic patients [8]. The blood glucose level depends on the dynamic balance between glucose consumption and production. Glucose production mainly comes from gluconeogenesis and hepatic glycogen decomposition, while glucose consumption partly depends on the utilization of peripheral tissues [54]. The liver is the main organ that maintains the balance of glucose metabolism and plays a core role in the pathogenesis of diabetes [44]. Phosphoinositide 3-kinase (PI3K)/Akt is the key pathway for the liver’s response to insulin [55]. Upon insulin engagement of its cognate receptor, PI3K is recruited to insulin receptor substrates (IRSs), which activates PI3K activity. PI3K activation produces phosphorylated phosphatidylinositol 3 (PIP3) at the plasma membrane, which binds to the intracellular PH domain-containing signaling proteins Akt and PDK1, prompting PDK1 to phosphorylate Akt proteins leading to their activation, which in turn regulates downstream proteins. Akt further phosphorylates and deactivates its target genes and signals to multiple downstream pathways to control the glucose level through gluconeogenesis and/or glycogen synthesis (Figure 1). One of the key target genes of Akt is glycogen synthase kinase-3 (GSK3), which is phosphorylated by Akt and thereby the phosphorylation of glycogen synthase (GS), resulting in the inactivation of GS activity and reduction of glycogen synthesis. Akt also blocks gluconeogenesis by promoting the phosphorylation and degradation of forkhead transcription factor 1 (foxo1) and downregulating the expression of phosphoenolpyruvate carboxykinase (PEPCK) and glucose-6-phosphatase (G6Pase), which are the rate-limiting enzymes [56] (Figure 1).

Research studies have revealed that enhancing the glucose catabolism may be another approach to treating diabetes [44]. Weier et al. demonstrated that the activation of M2-type pyruvate kinase (PKM2), a rate-limiting enzyme of glycolysis and catalyzing phosphoenolpyruvate (PEP) to pyruvate, restores mitochondrial function by increasing glucose metabolic flux, inhibiting the production of toxic glucose metabolites, and inducing mitochondrial biogenesis, thereby preventing diabetic nephropathy [57]. Articles published on cell metabolism showed that the activation of pyruvate kinase improved diabetes, indicating that the targeted inhibition of pyruvate kinase may be a new approach to diabetes treatment. In a study by Abudukadier et al. [58], the authors demonstrated that pyruvate kinase (PK) activators promoted the PEP cycle, thereby improving islet function and metabolic homeostasis. Apart from improving insulin secretion, acute PK activation can shorten gluconeogenesis, suppress endogenous glucose production, and accelerate erythrocyte glucose consumption. In line with this study, Lewandowski et al. [59] indicated that PK (rather than oxidative phosphorylation) triggers ATP/ADP to close the KATP channel of cells to initiate insulin secretion. The small-molecule activator of PK effectively promotes insulin secretion by converting mitochondria from oxidative phosphorylation to phosphoenolpyruvate biosynthesis. Overall, the PK activator enhanced the ability of insulin secretion but also consolidated glycolysis without increasing glucose oxidation, suggesting a potential therapeutic approach for diabetes based on PK activation.

### 5.1. Glucose Transporters

Glucose transporters (GLUTs), a member of the major facilitator superfamily of membrane transporters, are present in most mammalian cells and promote the transport of glucose across the plasma membrane [60]. Each isoform of glucose transporters plays a specific role in glucose metabolism. There are fourteen GLUT proteins expressed in humans [61], but at least six GLUT proteins are detected in the human liver: GLUT1, GLUT2 (also known as SLC2A2), GLUT5, GLUT8, GLUT9, and GLUT10 [61,62].

GLUT2 is an important glucose sensor encoded by SLC2A2 [63]. It is not only the most important glucose transporter in hepatocytes and is expressed in every hepatocyte but also widely exists in the pancreas and central nervous system [64,65]. GLUT2 plays an irreplaceable role in controlling glucose transport. Rémy et al. found that the loss of GLUT2 led to abnormal glycogen storage and that this abnormality is associated with glucose-6-phosphate levels [66]. Fanconi–Bickel (FBS) syndrome is a rare glycogen accumulation deficiency caused by SLC2A2 mutation. FBS patients present with hepatomegaly, postprandial hyperglycemia, and fasting hypoglycemia [67].

Studies have shown that the expression of GLUT8 in the liver of diabetic mice is regulated by insulin, suggesting that GLUT8 may be related to glucose homeostasis [68]. GLUT9 mainly transports uric acid with a high affinity [68]. Keembiyehetty et al. found that GLUT9 expression increased significantly in diabetic mice [69]. Although GLUT10 is widely expressed, its specific function in the liver remains unclear [63]. The expression levels of GLUT1 and GLUT5 are low in a healthy liver. GLUT1 was only expressed in a row of hepatocytes adjacent to the terminal hepatic vein [70]. However, in fasting and diabetes, the expression of GLUT1 is upregulated in certain hepatocytes that normally do not express the gene. Studies have shown that this increase is not related to blood glucose levels but to low circulating insulin levels [71]. GLUT5 expression is species-specific and only transports fructose. A high-fructose diet is associated with insulin resistance [72], but it is worth noting that GLUT5 mainly acts on fructose transport in the small intestine and may also play a secondary role in the liver [73,74].

In addition, sodium-glucose co-transporters (SGLTs) have received much attention in recent years for their therapeutic potential in diabetes. SGLT1 transporter proteins are more widely distributed in the intestine, heart and lung. SGLT1 is the main transporter protein responsible for glucose absorption in the intestine, and the inhibition of SGLT1 in the intestine leads to glucose-galactose malabsorption. The role of SGLT1 in intestinal and renal glucose transport makes the transporter protein a potential target for anti-hyperglycemic therapy. Decreased intestinal SGLT1 improves glucose homeostasis by inhibiting intestinal glucose uptake and inducing the sustained release of incretin. The increased expression of SGLT2, and the development of glycosuria, which are also classic sign of diabetes. SGLT2 transporter protein is located in the kidney, pancreatic alpha cells, and other tissues. Increased SGLT2-mediated glucose and sodium reabsorption in the kidney blocks urinary glucose excretion under high glucose and exacerbates the diabetic process. SGLT2 inhibition in pancreatic alpha cells stimulates glucagon secretion, which antagonizes the hypoglycemic effect of SGLT2 inhibitors by increasing hepatic glucose production. SGLT2 inhibition in type 2 diabetic patients reduces hyperglycemia and improves insulin secretion from beta cells and peripheral insulin sensitivity.

### 5.2. Glycogen Synthesis

Glycogen is a highly efficient storage form of glucose, and the benefit of this conversion form reaches 97% [75]. When the energy supply is insufficient, glycogen is immediately broken down into glucose to provide ATP [76]. Different from the continuous contraction energy supply of glycogen in muscle, glycogen in the liver is often used to maintain a constant blood sugar level [77]. The deficiency of insulin-mediated glycogen synthesis is one of the main causes of type 2 diabetes [78]. Xirouchaki et al. used the Cre/loxP system to generate muscle-specific gys1 knockout (gys1-KO) mice and found that under insulin stimulation, gys1-KO mice exhibited reduced muscle glucose uptake, peripheral insulin resistance, and exercise and endurance capacity, demonstrating that glycogen deficiency led to an impaired glucose metabolism and decreased exercise capacity [79].

In glycogen synthesis, the donor of glycosyl is uridine diphosphate glucose (UDPG), which is more active than glucose. Glycogen synthase catalyzes the formation of an α 1-4 glucosidic bond between the carbon atom of glucose molecule 1 on the UDPG molecule and the hydroxyl group of the fourth carbon atom of the glucose residue at a non-reducing terminal of glycogen [80]. Glycogen synthase, an important enzyme in glycogen synthesis, exists in two forms under physiological conditions: phosphorylated inactive glycogen synthase b and dephosphorylated active glycogen synthase a, which can be transformed into each other under the action of phosphoprotein phosphatase-1 (PP1) in the liver [81]. Meanwhile, GSK3 is a strong inhibitor of glycogen synthase, and glucose-6-phosphate is the sensor of this enzyme. A high concentration of glucose-6-phosphate activates glycogen synthase b in resting muscle and stimulates glycogen synthesis [82]. Studies have showed that glucose and insulin activate PP1 to stimulate glycogen synthesis, while glucagon and epinephrine inhibit its activity [83]. When eating, insulin activates Akt, inactivates GSK3, and increases the activity of PP1 by inhibiting GSK3, leading to the activation of glycogen synthase [84,85].

G protein coupled receptor (GPCR) on the cell surface also regulate blood glucose concentration in response to various of hormone signals, including the cAMP-PKA signal pathway. During fasting, the cAMP level increases, and cAMP-dependent protein kinase A (PKA) is activated [86]. The active PKA first phosphorylates glycogen phosphorylase kinase (GPK) for activation, which, in turn, activates glycogen phosphorylase (GP). Then the activated GP stimulates glycogen decomposition, and the activated PKA inhibits glycogen synthesis by phosphorylating GS. On the other hand, the activated PKA also activates phosphoprotein phosphatase inhibitor protein (IP), which inactivates phosphoprotein phosphatase (PP) to constrain glycogen synthesis and promote glycogen decomposition.

Glucokinase (GCK) is highly regulated for glycogen synthesis in the liver, but it is active in the cytoplasmic matrix [87]. Several studies have shown that GCK heterozygous inactivating mutations can lead to type 2 diabetes. Activated PKA also inhibits the transcription of the GCK gene, preventing the dissociation of GCK from glucokinase regulatory protein (GCKR) and GCK translocation to the cytoplasmic matrix [88,89,90]. GCKR mediates the activity of GCK, which is the rate limiting factor of GCK. Fernandes et al. found that the intron variation in GCKR gene was associated with 12 kinds of glycerides, 19 kinds of glycerophospholipids, and other lipids [91]. Insulin stimulates the transcription and translocation of GCK through Akt. Activated phosphodiesterase 3B reduces the intracellular level of cAMP, thereby inhibiting the cAMP-PKA pathway [92].

### 5.3. Gluconeogenesis and Upstream Transcription Factors

Gluconeogenesis refers to the synthesis of glucose from non-sugar substances as precursors. Gluconeogenesis is an indispensable metabolic pathway for human beings and other animals. When the body is in a state of hunger or strenuous exercise, the conversion of non-sugar substances into glucose is urgently needed [93].

Gluconeogenesis begins with saccharogenic amino acids or lactate, which is converted into pyruvate or glycogenic amino acids via lactate dehydrogenase to enter the tricarboxylic acid cycle to produce phosphoenol or pyruvate. Then pyruvate enters the mitochondria to form oxaloacetic acid under the catalysis of pyruvate carboxylase. Acetyl-CoA is a powerful allosteric activator of the enzyme, and the enzyme is inhibited by ADP [94]. Generally, oxaloacetic acid crosses the mitochondrial membrane through the formation of malic acid. Oxaloacetic acid is phosphorylated to PEP in the presence of PEPCK, the rate-limiting enzyme of gluconeogenesis pathway whose transcription is regulated by various factors, including glucagon and insulin. After several reverse steps of glycolysis, PEP is converted to fructose 1,6-diphosphate that is catalyzed by fructose 1, 6-diphosphatase, and that phosphate bond at the C1 position is hydrolyzed to form fructose 6-phosphate. In addition, citric acid stimulates fructose 1,6-diphosphatase and accelerates gluconeogenesis. Fructose-6-phosphate forms glucose-6-phosphate (G6P) through glucose phosphate isomerase, which is finally transferred to the endoplasmic reticulum to form free glucose and inorganic phosphate under the hydrolysis of glucose-6-phosphatase [95]. Fructose 2,6-diphosphate has a synergistic regulatory effect on fructose phosphokinase and fructose 1,6-diphosphatase which inhibits the activity of fructose 1,6-diphosphatase [96]. During starvation, low levels of fructose 1,6-diphosphatase promote the gluconeogenesis pathway [97]. Studies have shown that transcription factors often affect the entire pathway and reaction process of gluconeogenesis [98]. These transcriptional regulatory factors are likely to be pharmacological targets for the treatment of diabetes (Table 1).

**PGC-1α:** Peroxisome proliferator activated receptor γ coactivator 1 α (PGC-1α) is a central regulator of cellular metabolism [99]. PGC-1α is not only the main regulator of mitochondrial biogenesis and function but is also the activator of mitochondrial uncoupling [100]. Studies have shown that PGC-1α is associated with diabetes, obesity, neurodegeneration, and cardiovascular disease [101]. In the liver, PGC-1α is the main inducer of gluconeogenesis. During fasting, the PGC-1α level is significantly increased to stimulate gluconeogenesis [99]. PGC-1α interacts with hepatic nuclear factor 4α (HNF-4α) or foxo1, resulting in the transcription of PEPCK and G6Pase [102]. PGC-1α is also a direct transcriptional target of CREB [103]. CREB regulated transcription coactivator 2 (CRTC2) drives the expression of PGC-1α coactivators through its nuclear translocation and binding to CREB to control hepatic gluconeogenesis [104]. Besseiche et al. revealed that the overexpression of PGC-1α in a mouse fetal islet β cell resulted in a decreased number, weight, and function of islet β cells [105]. Additionally, the overexpression of PGC-1α in MIN6 cells increased mitochondrial oxidative stress, activated the AMPK signal pathway, and increased the level of AMPK phosphorylation, all of which inhibited insulin secretion [105]. Sawada et al. subjected isolated mouse skeletal muscle vascular endothelial cells to high concentrations of glucose (25 mmol/L) and physiological concentrations of glucose (5.5 mmol/L). They found that PGC-1α expression decreased rapidly within 4; otherwise, it significantly increased, suggesting that high glucose induced PGC-1α expression in skeletal muscle vascular endothelial cells [106]. Further studies revealed that PGC-1α overexpression strongly inhibited the production of vascular growth factor, inhibiting neovascularization in patients with diabetes [107]. Other reports have shown that PGC-1α gene knockout led to a reduction in the heterogeneous flux of glucose produced by PEP, thereby reducing glucose production. In skeletal muscle, PGC-1α activates the expression of glucose transporter protein 4 through the activation of the MEF2 transcription factor, which increases the uptake of glucose by these cells [108]. The increase in intracellular glucose concentration is accompanied by a decrease in glycolysis and an increase in glycogen storage. Indeed, the induction of PGC-1α leads to pyruvate dehydrogenase 4 (PDK4) expression via ERRα. This suggests that PGC-1α inhibits glucose oxidation and promotes glycogen synthesis.

**HNF-4α:** Hepatocyte nuclear factor 4α (HNF-4α) is a highly conserved member of the nuclear hormone receptor super-family (NR2A1), which is normally expressed in the pancreas, intestine, liver, and kidney and plays an important role in the regulation of liver and intestinal epithelial cell metabolism, cell connection, and the early embryonic development of the liver [109,110,111]. Moreover, HNF-4α is required for the expression of many genes in key metabolic pathways, such as control of gluconeogenesis and fatty acid transport [112]. In the liver, HNF-4α is well known for its role as the major regulator of liver-specific gene expression. In addition, genetic variation of HNF-4α is associated with many human diseases, including ulcerative colitis, colon cancer, adult diabetes, cirrhosis, and hepatocellular carcinoma [113,114,115]. It has been shown that HNF-4α deletion affects glucose transport, and glycolysis by affecting the expression of genes encoding GLUT-2, aldolase B, and L-PK, leading to abnormal insulin secretion [116]. HNF-4α is also an important nuclear transcription factor that regulates PEPCK transcription [112]. KooSH et al. found that dephosphorylated TORC2 binds to CREB transcription factor in the nucleus and stimulates the expression of PGC-1α. PGC-1α further induces HNF-4α to bind to the cis-element of its promoter, promoting the expression of key enzymes such as PEPCK and G6Pase and ultimately stimulating gluconeogenesis [117]. HNF-4α also regulates the main function of the liver by directly binding to the response elements of adjacent target genes and guide their expression. Wei et al. have confirmed that HNF-4α could bind to the miR-122 promoter region through the DR-I element (a binding site of HNF-4α), and positively regulate miR-122 expression in mice liver, regulating gluconeogenesis and lipid metabolism [118]. However, studies have also shown that post-translational modification regulated the activity of HNF-4α, including acetylation and ubiquitin. Bi et al. found that cholesterol sulfate (CS) and cholesterol sulfonyl transferase (SULT2B1b) inhibited gluconeogenesis by targeting HNF-4α in Hepa1-6 cells and transgenic mice [119]. Knockdown of Sult2B1b from primary mouse hepatocytes revealed an enhanced stimulation of gluconeogenesis by HNF-4α, increased expression of G6pase and PEPCK, and elevated fasting glucose levels in Sult2B1b^-/-^ mice [119]. Further studies showed that this change in activity was the result of increased HNF-4α acetylation through the inhibition of the HNF-4α deacetylase Sirt1. Mechanistically, CS and SULT2B1b inhibit gluconeogenesis by inhibiting the acetylation of the gluconeogenic factor HNF-4α and interfering with the nuclear translocation of HNF-4α [119].

**Foxo:** Foxo proteins are an evolutionarily conserved metabolic and stress response transcription factor. Mammalian foxo is divided into four distinct subtypes: foxo1, foxo3, foxo4, and foxo6, which control different cellular functions [120]. Foxo proteins play important roles in glycolipid metabolism, apoptosis, autophagy, the inhibition of the cell cycle, stress resistance, DNA repair, angiogenesis, inflammation, immune response, pluripotency, and differentiation [120]. Mutations in foxo genes or the abnormal expression of foxo proteins is associated with metabolic diseases, cancers, or lifespan changes in humans and animals [121,122]. During fasting, foxo protein promotes the gluconeogenesis of non-carbohydrate precursors by inducing the expression of the glucose isomerase G6Pase, fructose-1,6-diphosphatase, and PEPCK [120,123,124]. Studies have shown that foxo1 regulates glucose homeostasis [112,120]. The loss of foxo1 decreases hepatic gluconeogenesis [102]. In diabetic patients, insulin deficiency or insulin resistance leads to excessive activation of foxo1, which increases hepatic gluconeogenesis [125]. Nakae et al. found that foxo1 drives the expression of important genes for gluconeogenesis, such as G6PC [126]. Zhang et al. have shown that foxo1 protein promotes hepatic glucose production by mediating the expression of genes encoding important enzymes in the gluconeogenesis process, which, in turn, stimulates the release of glucagon from the pancreas and increases blood glucose [127]. Yan et al. found that the increased expression of lncRNA Gomafu also resulted in elevated foxo1 expression via miR-139, ultimately leading to the inappropriate activation of glycoisomerization [128]. Other studies have revealed that the phosphorylation of foxo1 leads to its inactivation in hepatocytes. Phosphorylated foxo1 binds to the core promoter Sin3a of glucokinase, which inhibits the expression of glucokinase gene and glucose production [129]. The initiation of the PI3K/AKT/Foxo1 pathway leads to the direct interaction and binding of phosphorylated foxo1 to peroxisome proliferator-activated receptor-γ coactivator-1, which inhibits the expression of gluconeogenesis. Additionally, insulin inhibits hepatic gluconeogenesis by activating the PI3K/Akt signaling pathway to phosphorylate and inactivate foxo1 [112]. Liu et al. found that iris reduced gluconeogenesis by downregulating PI3K/Akt/Foxo1-mediated PEPCK and G6Pase compared with the model group, thereby improving glucose homeostasis in STZ/HFD mice [122]. Foxo1 also contributes to the regulation of glucose in skeletal muscle [130]. Under the condition of chronic starvation, increased foxo1 expression in muscle binds to the promoter of pyruvate dehydrogenase kinase-4 gene, resulting in the conversion of pyruvate to acetyl-CoA and accelerating glucose consumption [131]. These results suggest that the inhibition of foxo1 is considered to be a therapeutic strategy for diabetes. In addition, there is evidence that other foxo proteins are also involved in this process. Glucose tolerance and insulin sensitivity were significantly increased in foxo1, foxo3, and foxo4 knockout mice compared to foxo1 knockout mice alone, suggesting that foxo3 and foxo4 contribute to the control of gluconeogenesis. Kim et al. found that increased hepatic glucose output in foxo6 transgenic mice, while hepatic foxo6 deficiency led to fasting hypoglycemia, suggesting that foxo6 is also involved in the regulation of gluconeogenesis [132]. These suggest that foxos play a key role in the treatment of diabetes.

## 6. Insulin-Mediated Signaling Pathway in Diabetes

Insulin performs all its possible physiological functions by binding to the insulin receptors on the plasma membrane of target cells. Insulin receptors are the main hub of the insulin signaling pathway which mediate the cellular function of insulin. An insulin receptor is a heterotetramer (α2β2) protein consisting of two α subunits and two β subunits connected through disulfide bonds. The two α subunits have insulin binding sites outside the plasma membrane, while the two β subunits are transmembrane proteins with kinase activity and signal transduction [133]. Insulin activates cascade phosphorylation upon binding to the ligands of insulin receptors on target tissue cell membranes. One is self-phosphorylation: the conformation of insulin receptors changes after insulin binding and the insulin receptors’ tyrosine kinase is activated; insulin receptors are activated after self-phosphorylation. Secondly, the downstream insulin receptor substrate proteins (IRSs) are phosphorylated, including IRS1-6, Shc, and Gab1. As early as 1976, Kahn et al. first suggested that insulin receptor abnormalities could cause diabetes [134]. A long-term study of a population with a high prevalence of type 2 diabetes also showed that insulin resistance preceded the onset of type 2 diabetes and was more severe in patients with type 2 diabetes who had insulin receptor variants than in those who did not [135]. Mice with knocked-out IRS-1 and IRS-2 genes in the liver exhibited significant signs of hyperglycemia, hyperinsulinemia, hyperlipidemia, and insulin resistance [136]. IRSs are an important mediator of insulin signal transduction, which activates insulin signal pathways, resulting in cell survival, differentiation metabolism, proliferation, senescence, glucose metabolism, adipogenesis, and a range of other biological effects [137,138,139,140]. Insulin receptors regulate metabolism mainly through two cellular signal cascades: (1) the mitogen-activated protein kinase (MAPK) pathway, directly controlling insulin-mediated metabolism through a variety of different mechanisms [141,142]; additionally, MAPKs also regulate gene expression; (2) the PI3K/Akt pathway, regulating the effects of insulin on acute cellular metabolism and cell growth and differentiation [143].

### 6.1. The MAPK Pathway Regulates Insulin-Mediated Metabolism

Currently, five different groups of MAPK have been identified in mammals: extracellular signal regulated kinases (ERKs) 1 and 2 (ERK1/2), c-Jun N-terminal kinases (JNKs) 1, 2 and 3 (JNK1/2/3), p38 α/β/δ/γ, ERKs 3 and 4 (ERK3/4), and ERK5 (also known as BMK1 or MAPK7) [144,145,146]. The JNK and ERK pathways play a crucial role in the development of obesity and insulin resistance. ERK pathway mediates the inflammatory-cytokine-induced downregulation of IRS1 expression [147]. ERK1/2 also catalyzes the phosphorylation of nuclear transcription factors, such as Ets, Elk, and c-Fos. The inflammatory cytokines, such as TNF)-α and interleukin (IL)-1β, and cellular stress (genotoxicity, permeability, hypoxia, or oxidative stress) are related with p38 and JNK signaling pathways. Ryan et al. confirmed that saturated fatty acids accumulated in obesity contribute to the activation of JNK [148]. In the context of obesity and type 2 diabetes, metabolic and inflammatory stress increase the activities of JNK and ERK in insulin target tissues, and these kinases induce serine phosphorylation of IRS1 or IRS2, whereas uncontrolled IRS serine phosphorylation promotes insulin resistance [149]. Hirosumi et al. found that high-fat-diet-fed JNK1^-/-^ mice not only attenuated weight gain, but also decreased insulin resistance and IRS1 serine phosphorylation and improved glucose tolerance [150]. Using a mouse model expressing Cre recombinase under the control of the albumin promoter, Sabio et al. selectively knocked out JNK1 from hepatocytes and found that hepatocyte-specific deletion mice with JNK1 did not prevent hepatic insulin resistance and improve glucose tolerance in high-fat-diet mice. In contrast, a JNK1 deficiency in adipose tissue decreased IL-6 secretion from the adipose tissue and inhibited high-fat-diet-induced hepatic insulin resistance [151]. Therefore, high JNK1 activity in adipose tissue leads to the imbalance of adipose cytokines and promotes insulin resistance [151]. However, Bost et al. found that ERK1-deficient mice were resistant to obesity and insulin resistance, immune to insulin resistance, and had a higher postprandial metabolic rate [152].

### 6.2. The PI3K/Akt Pathway Was Involved in Insulin Resistance

The PI3K/Akt signaling pathway is one of the major pathways of insulin signaling. Akt, a serine/threonine protein kinase, is a very important signaling molecule in the insulin-signaling pathway and is involved in mediating several cellular processes, including glucolipid metabolism, protein synthesis, cell proliferation, and apoptotic transcription. Andreelli et al. found that insulin increased the expression of P85 α-phosphatidylinositol-3-kinase mRNA in muscle [153]. Kadowaki et al. showed that PI3K knockout mice developed insulin resistance and diabetes [154]. Jiang et al. used siRNA to selectively inhibit the expression of Akt protein kinase in adipocytes and block the PI3K/Akt signal transduction pathway, resulting in insulin resistance in adipocytes [155]. By studying three subtypes of Pkb-specific deficient mice, Buzzi et al. found that the Pkb-α^-/-^ mice had significantly lower blood glucose, higher serum glucagon concentrations, and increased insulin sensitivity [156]. Further studies showed that overexpression of the IRS2-specific activation of AKT1 increased the number of β cells and insulin secretion in pancreatic islet cells [156]. A study by Cho et al. revealed that Akt2 knockout mice had reduced insulin sensitivity in the liver and skeletal muscle, as evidenced by reduced hepatic glycogen synthesis and reduced glucose uptake and utilization in skeletal muscle [157]. PDKl is a serine/threonine protein kinase that phosphorylates conserved AGC protein kinases, such as protein kinase B (PKB)/Akt, P70 ribosomal S6 kinase (S6K), serum- and glucocorticoid-induced protein kinase (SGK), and protein kinase C (PKC) isoforms, which play an important role in regulating physiological processes related to cell growth, proliferation, survival, and metabolism [158]. Mora et al. used a CRE recombinase/loxP technique to make mice hepatocytes lack PDK1 expression, resulting in poor glucose tolerance and 10-fold lower liver glycogen levels compared to controls [159]. Hashimoto et al. found that islet volume was reduced in mice specifically lacking PDK1 in their pancreatic β-cells, leading to diabetes. These results suggest that PDK1 plays an important role in maintaining pancreatic cell numbers and glucose homeostasis [160]. The above studies suggest that type 2 diabetes is closely associated with the PI3K/Akt signaling pathway and that a decrease in the level and activity of any substance in this pathway leads to the disruption of normal physiological insulin metabolism.

## 7. Roles of Long Non-Coding RNA in Diabetes and Its Complications

**Figure 2:**
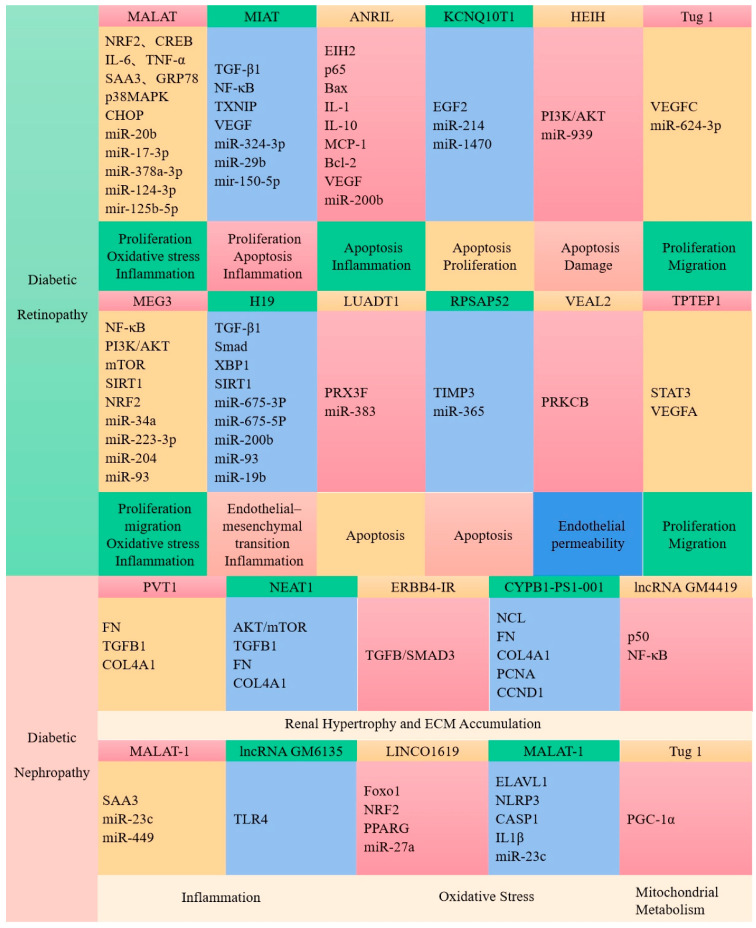
LncRNAs are closely associated with the development of diabetic complications.

Non-coding RNA (ncRNA) is a functional RNA molecule that is not translated into protein. NcRNA family members include microRNAs (miRNAs), small interfering RNAs (siRNAs), piwi-interacting RNAs (piRNAs), small nucleolar RNAs (snoRNAs), small nuclear ribonucleic acids (snRNAs), extracellular RNAs (exRNAs), small cajal-body-specific RNAs (scaRNAs), long ncRNAs (lncRNAs), and nonlinear cyclic RNAs [161]. Studies have shown that lncRNAs are closely associated with abnormal blood glucose levels and insulin resistance in patients with type 2 diabetes and are considered important participants in the development of diabetes and diabetic complications (Figure 2), suggesting that the potential of lncRNAs as therapeutic targets for clinical applications in the management of diabetes [162,163,164,165].

### 7.1. LncRNAs Regulate Glucose Metabolism in Diabetes

The typical feature of diabetes is the disturbance of glucose metabolism. It has been established that lncRNAs play an important role in the process of glucose metabolism in diabetes. Some lncRNAs participate in hepatic insulin resistance by regulating the expression and activity of foxo1. In high-fat-diet mice, ob/ob mice, and palmitate-, oleate-, or linoleate-treated hepatocytes, elevated levels of the lncRNA MEG3 correlated with increased G6PC, PEPCK, and foxo1 levels, which resulted in enhanced gluconeogenesis and impaired insulin-stimulated glycogen accumulation in hepatocytes, manifesting as hepatic insulin resistance [162]. An increase in the lncRNA Gomafu led to elevated foxo1 and gluconeogenic protein expression through sponge miR-139 [128]. MEG3 knockdown improved glucose tolerance in high-fat-diet and ob/ob mice [166]. The upregulation of MEG3 increased hepatic gluconeogenesis and inhibited glycogen synthesis in mouse hepatocytes through histone acetylation [166]. LncLGR inhibited glucokinase activity by interacting with hnRNPL, thereby blocking hepatic glycogen synthesis [167]. H19 was significantly reduced in the liver of db/db mice. Silencing H19 enhanced gluconeogenic gene levels and glucose output in human hepatoma and primary hepatocytes [168]. The knockout of SAR1 enhanced insulin sensitivity, improved glucose tolerance, and suppressed obesity in mice. SAR increased insulin-sensitive glucose uptake by activating the Akt pathway and the phosphorylation of foxo1 [169]. These results suggest that lncRNAs are involved in the process of glucose metabolism in diabetes.

### 7.2. Roles of LncRNAs in Islet

In addition, the abnormal expression of lncRNAs in the pancreatic β cells of diabetic patients is usually located near islet-specific chromatin or at diabetes susceptibility loci [170]. Changes in pancreatic islets are important indicators of diabetic pathology. In 2012, Ding et al. found that the lncRNA H19 is involved in the intergenerational transmission of gestational diabetes, resulting in impaired islet structure and function [171]. Yin et al. demonstrated that silencing the lncRNA TUG1 significantly raised the apoptosis of pancreatic β cells, decreased insulin secretion, and led to elevated fasting blood glucose [172]. It has also been reported that the silencing of the lncRNA MEG3 resulted in impaired glucose tolerance, decreased insulin secretion, and fewer insulin-positive cells, indicating that MEG3 may be a novel β cells regulator [166]. Xu et al. reported that the lncRNA SRA has a positive effect on promoting insulin sensitivity and insulin-stimulated glucose uptake by adipocytes [168]. The overexpression of the lncRNA SRA in ST2 adipocytes suppressed the expression of negative regulators of insulin sensitivity (cytokine signal transduction factor-1 and -3 repressors) while increasing the expression of positive regulators (SH3 structural domain containing 1) [168]. In addition, the LncRNA SRA enhances insulin function through Akt and foxo1 signaling pathways [168]. These studies suggest that lncRNAs play an important role in islet function.

### 7.3. LncRNAs Are Associated with Diabetic Complications

Apart from the roles in diabetes discussed above, lncRNAs are implicated in complications associated with diabetes. Diabetic retinopathy is one of the most common complications in diabetic patients which leads to visual impairment or loss [173]. Yan et al. found that 303 lncRNAs were abnormally expressed in the retinas of patients with early diabetic retinopathy, and an enrichment analysis showed that these lncRNAs were mainly involved in the MAPK signaling pathway, complement and coagulation cascade, chemokine signaling pathway, pyruvate metabolic pathway, and axon guidance signaling pathway [174]. Changes in the expression of the lncRNAs MALAT1 and MEG3 have been reported to be associated with diabetic retinopathy [174,175,176,177]. The downregulation of the expression of the ncRNA MALAT1 ameliorated streptozotocin-induced retinopathy in rats [177]. The lncRNA MEG3 was similarly downregulated in the retinas of streptozocin-induced diabetic mice [178]. Another major complication associated with diabetes is diabetic nephropathy. LncRNAs also play important role in the development of diabetic nephropathy and the accumulation of extracellular matrix (ECM) proteins [179]. Plasmacytoma variant translocation 1 (PVT1) was the first gene identified as a candidate for end-stage renal disease in type 2 diabetes. In patients with diabetic nephropathy, the expression of lncRNA PVT1 is increased, and the accumulation of ECM proteins in renal cells leads to increased fibrosis. Conversely, the downregulation of PVT1 reduced the accumulation of ECM [180,181]. Alvarez et al. showed that the overexpression of PVT1 dramatically enhanced the TGFB1, PAI-1, FN1 mRNA, and protein levels, while PVT1 knockdown resulted in the reduced expression of these molecules [180,181]. Yi et al. revealed that diabetic nephropathy is also associated with elevated levels of FN1 mRNA lncRNA Gm4419, acting through the interaction with NF-κb. Under the condition of hyperglycemia, the knockdown of FN1 mRNA lncRNA Gm4419 in mesangial cells inhibited cell proliferation and NF-κb expression [182]. Kato et al. found that in mesangial cells, TGF-β induces the expression of miR-192 and its host lncRNA CJ241444, leading to the activation of Akt and p300, resulting in mesangial hypertrophy [183]. In addition, diabetes also causes cardiovascular disease; however, lncRNAs are rarely directly associated with diabetic vascular complications [184]. The lncRNA MALAT1 was upregulated during cardiomyopathy, as shown by differential expression analysis in the heart tissue of normal and diabetic rats in reports by Zhang et al. [185,186]. The downregulation of FN1 mRNA lncRNA MALAT1 improved left ventricular systolic function via reducing myocardial information in diabetic rats [185,186]. Similarly, the overexpression of H19 in diabetic rats reduced oxidative stress and apoptosis and improved ventricular function [187]. These studies revealed that lncRNAs play an important role in the pathogenesis of diabetic complications.

## 8. The Disturbance of Intestinal Flora May Partly Give Rise to Diabetes

The gut microbiota is closely related to the development and progression of type 2 diabetes. An imbalance of intestinal flora in diabetic patients leads to the disruption of the diversity and stability of microflora. Larsen et al. showed that the levels of *Firmicutes* and *Clostridium* in type 2 diabetic patients were significantly reduced [188]. Qin et al. reported that the intestinal microbiome of type 2 diabetic patients had lower levels of *Faecalibacterium* and *Roseburia* (a butyrate producer) [189]. An increase in pathogenic bacteria and a decrease in butyrate-producing bacteria induced low-grade, chronic intestinal inflammation and triggered insulin resistance. Abnormal ratios of *Bacteroides*/*Firmicutes* are associated with increased intestinal permeability and the infiltration of bacterial byproducts through the leaky intestinal barrier, causing inflammation [190]. *Lactobacillus*, *Roseburia*, *Bacteroides*, and *Akkermansia* reduced the risk of diabetes by inhibiting pro-inflammatory factors and maintaining intestinal barrier integrity, improving glucose metabolism and insulin sensitivity [190]. Decreases in probiotics such as *Lactobacillus* and *Bifidobacterium* were strongly associated with abnormal glucose tolerance. Zhou et al. found that the relative abundance of *Ruminococcus*, *Allococcus*, and *Lactobacillus* was correlated with blood glucose levels in ZDF rats [191]. Yang et al. showed that genistein alleviated insulin resistance and inflammatory responses by regulating the abundance of *Bacteroides*, Prevotella, and *Helicobacter* in mice induced by a high-fat diet and streptozotocin [192]. A decrease in butyrate in type 2 diabetes affects glucose homeostasis and islet cell function. For example, butyrate affects glucose homeostasis by activating intestinal gluconeogenesis [193]. Increased intestinal butyrate production was associated with an improved insulin response after oral glucose testing. Butyrate improved insulin resistance, hyperglycemia, and hyperinsulinemia in obese/pre-diabetic mice [194]. It has also been shown that butyrate improved the progression of type 2 diabetes by maintaining the integrity of the intestinal epithelial barrier, promoting hepatic glycogen metabolism, and regulating mitochondrial function [195]. Propionate, another important short-chain fatty acid, not only promoted the release of GLP-43 and peptide YY through the activation of GPR1 to enhance insulin sensitivity, but also activated intestinal gluconeogenesis and maintained glucose and energy homeostasis through GPR41-dependent gut–brain circuits [195]. However, acetate improved reproductive system function by inhibiting HDAC5 and PCSK9 in female and male diabetic patients, respectively [195]. In conclusion, SCFAs play important role in the development of type 2 diabetes.

## 9. Sirtuins as a Novel Drug Target for Type 2 Diabetes

Sirtuins are a series of highly conserved NAD (+)-dependent deacetylases involved in various physiological and pathological activities in the body. SIRT1 reduced inflammation in adipose tissue and monocytes/macrophages and improved insulin resistance in type 2 diabetes. Decreased SIRT1 contributed to TNF-α-induced insulin resistance in skeletal muscle of patients with type 2 diabetes [196]. SIRT1 protected pancreatic β-cells from various toxic stresses by inhibiting NF-κB signaling [197]. The activation of SIRT1 may improve insulin resistance by accelerating fatty acid oxidation, the deacetylation of PGC-1α, and the activation of PPAR-α in skeletal muscle [198]. It has also been reported that SIRT1 is involved in glucose metabolism. During fasting, SIRT1 is activated and deacetylates CRTC2 to reduce the effects of glucagon [199]. SIRT1 overexpression in transgenic mice reduced hepatic glucose output and improved glucose tolerance [199]. SIRT1 induced gluconeogenesis-related genes by activating PGC-1α and FOXO1 through a deacetylation reaction [199]. SIRT1 also deacetylates STAT3, resulting in reduced STAT3 activity and the inhibition of gluconeogenesis [199].

SIRT2 is widely expressed in various tissues and plays an important role in various physiological processes that maintain metabolic homeostasis, including gluconeogenesis and insulin sensitivity. SIRT2 expression in PMCS is negatively correlated with insulin resistance. Lemos et al. reported that SIRT2 is downregulated in insulin-resistant hepatocytes and liver [200]. Overexpression of SIRT2 in insulin-resistant hepatocytes improved insulin sensitivity and reduced the production of ROS [198]. SIRT2 is involved in the regulation of gluconeogenesis through PEPCK. Jiang et al. showed that SIRT2 decreased the acetylation of PEPCK1 and the association between PEPCK1 and UBR5, increased the stability of PEPCK1, and promoted gluconeogenesis [201]. It has also been shown that SIRT2 inhibition not only increased the stability of GKRP protein and promoted the degradation of ALDOA but also inhibited glucose-stimulated insulin secretion and reduced glycolytic flux by regulating the Akt/GSK3β/β-linked protein pathway in β-cells [202].

Sirtuins also prevent diabetic nephropathy. SIRT1 deletion in podocytes of diabetic db/db mice led to the acetylation of NF-κB and the p65 subunit of STAT65 and increased urinary protein excretion and renal injury [203]. The targeted disruption of SIRT1 in the proximal tubules of diabetic nephropathy mice led to the ectopic expression of the tight junction protein claudin-1 in podocytes, resulting in albuminuria and renal impairment [204]. SIRT3 activation via G-protein-coupled bile acid receptors prevented oxidative stress and lipid accumulation in diabetic nephropathic mice [203].

## 10. Summary

Diabetes has become a global crisis that threatens the health and economy of the world. A more complete understanding of diabetes will enable people to take targeted preventive measures. A healthy lifestyle and social and medical support are also greatly significant for the prevention of diabetes and its complications. Studies have revealed that the regular intake of cereals can reduce the risk of chronic diseases, but further determination of the specific molecular mechanisms is still required. Meanwhile, the rational implementation and use of diabetes drugs is still a practical challenge for clinicians.

## Figures and Tables

**Table 1:** Role of important transcription factors upstream of gluconeogenesis in diabetes.

Transcription Factors	Related Processes	Related Genes/Proteins
PGC-1α	Hepatic insulin resistance; fatty acid oxidation	PPARα
	Hepatic gluconeogenesis	CREB, CRTC2
	Insulin secretion	AMPK signal pathway
	The uptake of glucose	MEF2
	Glycogen synthesis	PDK4
HNF4α	Gluconeogenesis	PGC-1α; CS; SULT2B1b
	Gluconeogenesis; lipid metabolism	DR-1 MiR-122
Foxo	Gluconeogenesis	G6Pase; PEPCK; PGC-1α; PI3K/AKT
	Gluconeogenesis	LncRNA Gomafu; miR-139
	Glucose consumption	PDK4
	Glucose production	Sin3a
